# Prevalence of osteoporosis in China: a meta-analysis and systematic review

**DOI:** 10.1186/s12889-016-3712-7

**Published:** 2016-10-03

**Authors:** Peng Chen, Zhanzhan Li, Yihe Hu

**Affiliations:** 1 Department of Orthopedic, Xiangya Hospital, Central South University, No.87, Xiangya Road, Kaifu District, Changsha City, Hunan Province 410008 China; 2 Department of Oncology, Xiangya Hospital, Central South University, Changsha, Hunan Province China

**Keywords:** Osteoporosis, Prevalence, Cross-sectional study, Meta-analysis, China

## Abstract

**Background:**

We conducted a systematic review and meta-analysis to obtain a reliable estimation of the prevalence of osteoporosis in China and to characterize its epidemiology.

**Methods:**

We identified relevant studies via a search of literature published from 2003 to October 2015 in the PubMed, Web of Science, China National Knowledge Infrastructure, Wanfang and Weipu databases. Both Chinese and WHO criteria were considered acceptable for the diagnosis of osteoporosis. Prevalence estimates were obtained using random effects models. Meta-regression analysis was used to explore the sources of heterogeneity, and publication bias was evaluated by visually inspecting funnel plots.

**Results:**

Overall, 69 articles were included in this study. An obvious increase in the prevalence of osteoporosis was identified over the past 12 years (prevalence of 14.94 % before 2008 and 27.96 % during the period spanning 2012–2015). The prevalence of osteoporosis was higher in females than in males (25.41 % vs. 15.33 %) and increased with age. Osteoporosis prevalence was higher in rural than in urban areas (20.87 % vs. 23.92 %) and higher in southern than in northern areas (23.17 % vs. 20.13 %). At present, the pooled prevalence of osteoporosis in people aged 50 years and older was more than twice the pooled prevalence identified in 2006 (34.65 % vs. 15.7 %). The application of different diagnostic criteria could have an impact on prevalence estimation (19.7 % vs. 29.3 %). Meta-regression suggested that study setting also influenced the estimation of point prevalence (*P* = 0.022).

**Conclusions:**

The prevalence of osteoporosis in China has increased over the past 12 years, affecting more than one-third of people aged 50 years and older. The prevalence of osteoporosis increased with age and was higher in females than in males. Prevention and control measures have become all the more important given the increase in osteoporosis prevalence, and three-step prevention programmes should be implemented.

**Electronic supplementary material:**

The online version of this article (doi:10.1186/s12889-016-3712-7) contains supplementary material, which is available to authorized users.

## Background

Osteoporosis is a metabolic disease associated with decreased bone strength and characterized by bone mass reduction, increased skeletal fragility and bone tissue structure deterioration [1]. Osteoporosis is the most frequent cause of bone fractures in the elderly, especially spine, proximal femur (hip), distal forearm, and proximal humerus fractures [2]. Typically, symptoms do not appear until the occurrence of a broken bone, and even minor stress may induce fractures when bone mineral density (BMD) is decreased. Chronic pain caused by osteoporosis has been found to severely interfere with normal activities [3]. In developed countries, the prevalence of osteoporosis ranges from 2 to 8 % among males and 9 to 38 % among females depending on the method of diagnosis [4]. It has been reported that approximately 2 million males and 8 million females above the age of 50 years in the United States have been diagnosed with osteoporosis, and 34 million people are estimated to have osteopenia [5]. Approximately 5.5 million men and 22 million women in Europe were affected by osteoporosis in 2010 [6]. Although the exact rates of osteoporosis remain unclear [7], this condition is becoming an urgent health concern worldwide. In addition to increasing activity restriction and the risk of fragility fractures, osteoporosis may increase the risk of hospitalization associated with some complications, and thus imposes a huge economic burden on the public health system [8]. Using hip fractures as an example, hip fractures have been estimated to be associated with a 10 to 20 % increase in mortality, and an estimated 25 % of people with hip fractures in the United States need long-term home nursing care [9]. The cost of each hip fracture was estimated to be $34,000 to 43,000, and the annual cost of all osteoporosis-related fractures is estimated to reach 18 billion dollars [10].

It is foreseeable that China may face a similarly severe health problem in the future. This is apparent due to two main reasons. One reason is the huge population that may result in a large population of people with osteoporosis in China. The other reason is that the ageing population may be associated with increased pension and healthcare costs, forcing large increases in public spending, as advanced age is one of the main risk factors for osteoporosis [11]. The most recent nationwide osteoporosis survey, conducted in 2006, showed that the prevalence rates of osteoporosis among those above the age of 50 years were 57.6 % in males and 64.6 % in females. Almost 10 years have passed since this survey. Previous studies have focused more on determining reference values of BMD and were conducted before 2008 [12, 13]. A reliable estimate of osteoporosis prevalence is necessary in countries such as China. This estimate may have a particularly practical significance in providing guidance for the control and prevention of osteoporosis. As China has a huge population, even modest progress in the preventive management of osteoporosis can significantly improve population level health outcomes. For these reasons, we conducted a systematic review and meta-analysis to reliably estimate the prevalence and characterize the epidemiology of osteoporosis in China.

## Methods

An ethical statement is not needed for this study because this is a meta-analysis and systematic review based on published studies. We conducted this meta-analysis and systematic review in accordance with the 2009 PRISMA (Preferred Reporting Items for Systematic Reviews and Meta-Analyses) guidelines [14].

### Search strategy

We conducted electronic searches of the PubMed, Web of Science, CNKI (China National Knowledge Infrastructure), Wanfang (Chinese) and Weipu (Chinese) databases to identify population-based studies that measured the prevalence of osteoporosis from inception until October 2015. These searches used free text and medical subject heading terms and combined osteoporosis-related keywords. Search terms included ‘osteoporosis’, ‘osteopenia’, ‘OP’, ‘bone mineral density’, ‘brittle-bone disease’, ‘bone’, ‘prevalence’, ‘cross-sectional’, ‘epidemiology survey or investigation’, ‘China’, and ‘Chinese’. Language of publication was restricted to English and Chinese. We also retrieved the reference lists of included articles and previous reviews to identify potential studies as comprehensively as possible. Studies were restricted to those evaluating the Chinese population.

### Criteria for inclusion

The inclusion criteria are listed as follows:

1. Study population: Participants were aged ≥15 years and included a representative sample of Chinese population or mixed population, and the study was conducted in a geographically defined or clinical setting.
2. Period: The time period of the study was restricted to the period from January 2003 to October 2015.
3. Study type: Data from cross-sectional studies or baseline investigations from prospective studies with defined osteoporosis diagnosis criterion were included (Additional file 1).
4. Information: Studies including metrics for sample size and directly and/or indirectly providing prevalence of osteoporosis with or without age-specific estimates were included.

### Criteria for exclusion

Studies conducted in a population with specific other diseases or occupations were excluded (connective tissue disease, gastrointestinal and nutritional diseases, endocrine and metabolic diseases, haematological system diseases, and a population working in an environment with lead, cadmium and aluminium). Studies with sample sizes smaller than 100 participants were excluded. Reviews, commentaries, and case reports were also excluded.

### Data extraction

Two investigators (CP and LZZ) independently extracted data using a standardized data collection sheet. Disagreements were resolved through discussion with team members. The following information was collected from each study: year of publication, year in which the study was conducted, first author, province, study design, area (northern or southern), region (urban or rural), minimum age of participants, number of osteoporosis cases and sample size, response rate of the survey, method of sample selection, source of sample, diagnostic criteria (World Health Organization (WHO) or Chinese), equipment used for BMD measurement, and study quality score. We also contacted the authors of an article if necessary. The outcome of interest was the prevalence rate of osteoporosis in different settings.

### Quality assessment

The quality of each included study was assessed using the quality assessment criteria for observational studies recommended by the Agency of Healthcare Research and Quality. These assessment criteria include 11 criteria with three potential responses: Yes, No and Unclear. Briefly, the 11 criterion are as follows: 1: Does the study define the information source of the survey? 2: Does the study report clear inclusion or exclusion criteria? 3: Does the study report the time period of patient inclusion? 4: Was the study population enrolled consecutively? 5: Does the study indicate whether the evaluators of subjective components were blind to other aspects of participant status? 6: Was any quality control conducted? 7) Does the study describe the excluded patients in detail? 8: Does the study indicate whether the models included adjustment for potential confounders? 9) How did the authors address missing data, if applicable? 10: Does the study summarize patient response rates and completeness of data collection? 11: Does the study describe follow-up and the estimated percentage of incomplete data? A maximum score of 11 was possible for each study. A score ≤5 points was considered as low quality [15].

### Statistical analysis

We estimated the prevalence rates of osteoporosis with 95 % confidence intervals (CIs) overall and by subgroup. The point prevalence rates were first transformed into arcsine square root transformed proportions. The transformed data were fitted for a random effects model using DerSimonian-Laird weights, and studies with 0 cases were still included in the overall analysis [16]. Heterogeneity across studies was examined using Cochran chi-square (*χ*^*2*^) tests. The classification of heterogeneity depended on the *I*^*2*^ statistic: < 25 % indicated a low level, 25–50 % indicated a moderate level, and >50 % indicated a high level of heterogeneity [17]. We adopted a random effects model to estimate the prevalence of osteoporosis and performed subgroup analyses by year of data collection (before 2008, 2009–2011, and 2012–2015), region (urban and rural), area (South and North China), age at onset (15–24, 25–35, 46–49, and 50- years), gender (female and male), age group (15-, 30-, 40-, 50-, 60-, 70-, 80- years) overall and separately for males and females, and diagnostic criteria (WHO vs. Chinese). The categorization of year of data collection was based on the distribution of the number of studies; age at onset was categorized based on the categorization and lack of specific ages within the included studies. To explore the main factors influencing prevalence estimation and sources of heterogeneity, we conducted meta-regression analysis including the following covariates: year of publication, year of data collection, female ratio (%), area, source, response rate, region, criterion, age at onset, and quality score. Publication bias was evaluated by inspecting Begg’s funnel plots with log prevalences and standard errors. Begg’s Test and Egger’s Test were also used for qualitative judgements of bias. *P* < 0.05 was considered statistically significant.

## Results

The preferred reporting items for this systematic review and meta-analysis are presented in Additional file 2.

### Study selection and characteristics

**Fig. 1:**
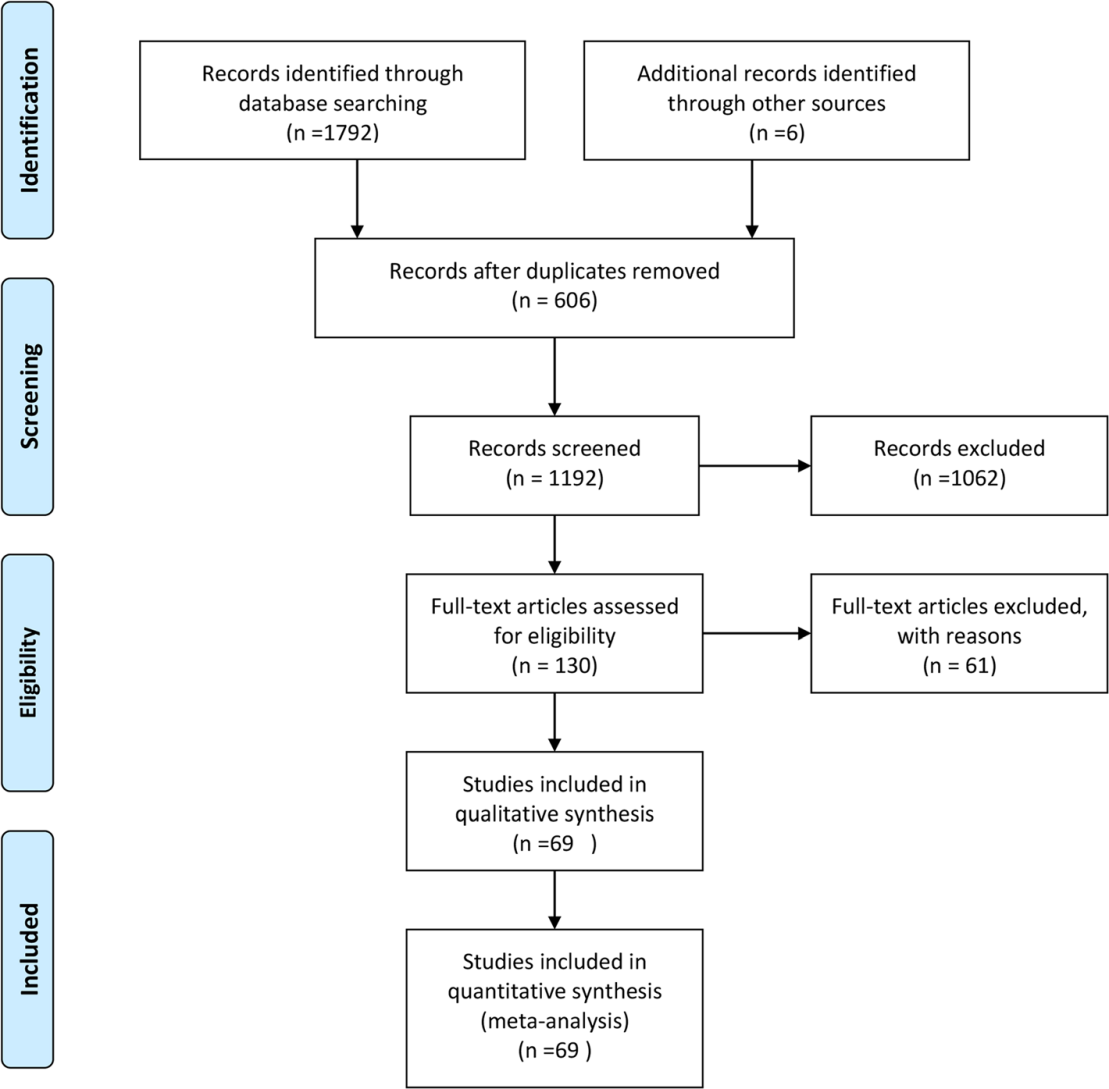
Flow diagram of included/excluded studies

The flow chart for study selection is presented in Fig. 1. In brief, our initial searches identified 1798 records. After title and abstract screening, 606 duplicates were removed, and 1062 records were excluded for different reasons; following this exclusion, 130 full-texts were assessed using the inclusion criteria. Finally, 69 studies (Additional file 3) were included in qualitative and quantitative synthesis.

The characteristics of the 69 included studies are shown in Additional file 4. The studies were published between 2003 and 2015. Of the 69 studies, 50 had been conducted in urban areas and 19 had been conducted in rural areas; 36 studies were conducted in South China and 33 were conducted in North China; 39 studies evaluated osteoporosis based on the WHO diagnostic criteria and 30 evaluated osteoporosis based on Chinese criteria; and 53 and 56 studies focused on males and females, respectively. The sample size per study ranged from 179 to 19,609, and the total population included in this meta-analysis was 184,100 participants, including 75,710 males and 108,390 females. The total number of included participants was not equal to the sum of numbers of males and females because some studies only provide total numbers and did not separate participants into males and females. The point prevalence of osteoporosis varied from 1 to 86 %. Except one cohort study, most studies had a cross-sectional design. The survey response rates were mostly above 90 %. The response rates ranged from 85.9 to 100.0 % with a mean of 96.6 %. The quality scores of four studies were less than five points because of insufficient sample sizes. The overall quality of the included studies was acceptable.

### Pooled prevalence rates of osteoporosis

#### Overall

**Fig. 2:**
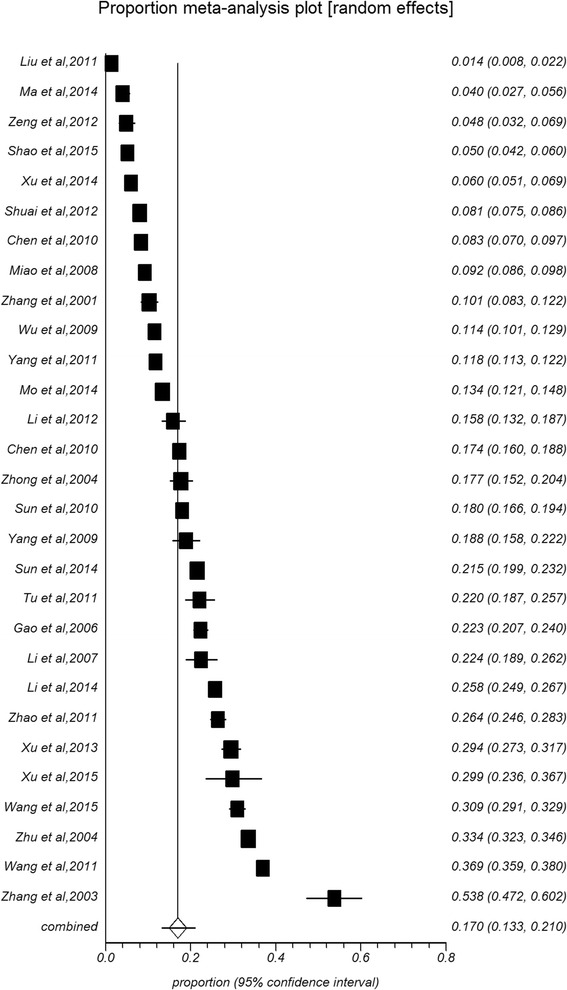
Forest plot of prevalence of osteoporosis for total people

The meta-analysis of the total prevalence estimates of studies evaluating participants with an onset age of 15–24 years (*n* = 29, *N* = 97,997) showed that the prevalence rate of osteoporosis was 16.96 % (95 % CI: 13.26–21.02 %, Fig. 2﻿, Table 1) with a high level of heterogeneity (99.6 %). The prevalence rates of osteoporosis at onset ages of 25–35, 46–39, and 50- years were 28.09 % (95 % CI: 18.11–39.32 %), 28.04 % (12.11–42.95 %) and 34.65 % (30.30–44.24 %), respectively. Thus, estimated prevalence rates increased with age.

**Table 1:** Prevalence of osteoporosis according to different items

Category	Subgroup	NO. of Studies	Prevalence *(95 % CI*)(%)	*N*	*I* ^*2*^ (%)	Publication bias	
						*P*(Begg) *P*(Egger)	
Study year	Before 2008	17	14.94 [10.65–19.8]	49824	99.7	0.228	0.493
	2009–2011	20	23.65 [17.57–30.35]	74250	99.8	0.003	0.055
	2012–2015	16	27.96 [19.90–36.81]	36106	99.5	0.128	0.100
Region	Urban	50	20.87 [17.05–24.96]	124462	99.7	0.623	0.050
	Rural	19	23.92 [17.17–31.39]	67226	99.8	0.093	0.056
Area	Southern	36	23.17 [18.29–28.44]	95814	99.7	0.003	0.000
	Northern	33	20.13 [15.62–25.06]	95874	98.7	0.559	0.343
Sex	Male	53	15.33 [11.80–19.21]	75710	99.5	0.105	0.379
	Female	56	25.41 [21.54–29.49]	108390	99.6	0.072	0.118
Onset age of study(y)	15–24	29	16.96 [13.26–21.02]	97997	99.6	0.053	0.035
	25–35	9	28.09 [18.11–39.32]	24122	99.7	0.119	0.069
	46–49	20	28.04 [12.11–42.95]	44500	99.7	0.028	0.015
	50-	11	34.65 [30.30–44.24]	24969	99.7	0.000	0.019
Age-specific group(y)	15-	27	2.40 [1.32–3.79]	7265	91.1	0.532	0.134
	30-	36	6.49 [4.52–8.77]	21541	97.4	0.004	0.157
	40-	49	8.88 [7.05–10.90]	43160	97.9	0.0128	0.000
	50-	51	19.57 [16.29–23.07]	41983	98.7	0.143	0.169
	60-	54	35.10 [29.71–40.42]	36453	99.1	0.172	0.256
	70-	45	43.48 [37.69–49.37]	19238	98.5	0.135	0.009
	80-	26	56.10 [46.44–65.52]	3827	97.2	0.827	0.027
Male(age)	15-	24	1.12 [0.51–1.97]	3586	73.5	0.001	0.069
	30-	30	3.43 [2.25–4.84]	9132	90.4	0.090	0.301
	40-	38	6.42 [4.78–8.27]	17055	94.8	0.755	0.240
	50-	38	11.64 [9.08–14.47]	14756	96.1	0.044	0.145
	60-	38	18.71 [14.90–22.84]	12028	96.7	0.260	0.001
	70-	33	28.6 [23.3–34.21]	6740	95.9	0.189	0.862
	80-	23	36.53 [27.71–45.82]	1421	91.9	0.601	0.452
Female(age)	15-	22	2.18 [1.06–3.68]	3433	84.4	0.524	0.017
	30-	29	6.84 [4.15–10.12]	11530	97.3	0.065	0.016
	40-	39	10.10 [7.59–12.92]	23318	97.6	0.037	0.304
	50-	39	23.85 [18.93–29.14]	21574	98.7	0.141	0.052
	60-	42	45.77 [38.38–53.25]	15226	98.8	0.124	0.323
	70-	34	58.26 [49.26–67.00]	7420	98.3	0.032	0.291
	80-	22	68.45 [57.83–78.17]	1208	93.2	0.069	0.676
Criteria for diagnostic	WHO	39	20.35 [16.01–25.07]	123900	99.7	0.090	0.159
	China	30	23.4 [18.56–28.77]	67698	99.6	0.047	0.235
Source of population	General	45	19.7 [16.0–22.90]	145985	99.6	0.673	0.467
	Hospital	24	29.3 [21.75–36.62]	45703	99.7	0.213	0.503

#### Study year

The meta-analysis results showed a general upward trend. The prevalence rates among studies with data collected before 2008, from 2009 to 2011, and from 2012 to 2015 were 14.94 % (95 % CI: 10.65–19.8 %), 23.65 % (17.57–30.35 %) and 27.96 % (19.90–36.81 %), respectively. These data indicated that osteoporosis prevalence slightly increased from 2009–2011 to 2012–2015; however, the prevalence during both of these time periods were obviously higher than the prevalence before 2008.

#### Sex- and age-specific groups

**Fig. 3:**
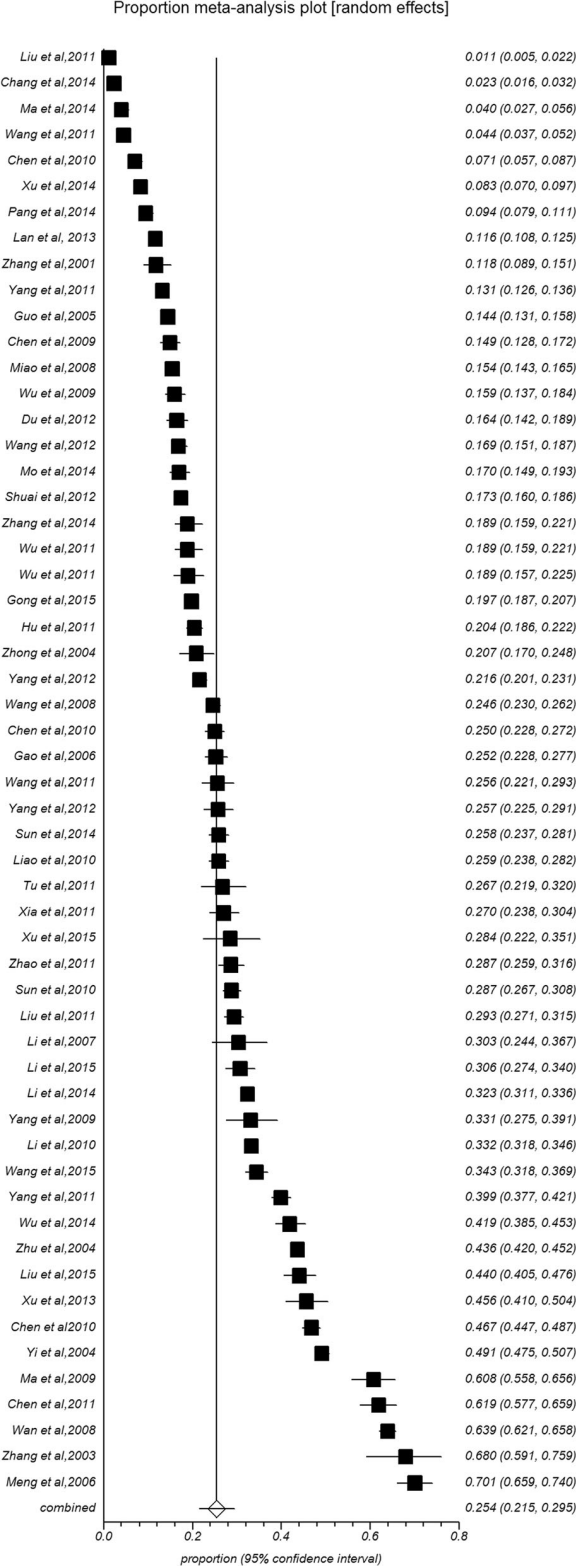
Forest plot of prevalence of osteoporosis for female

**Fig. 4:**
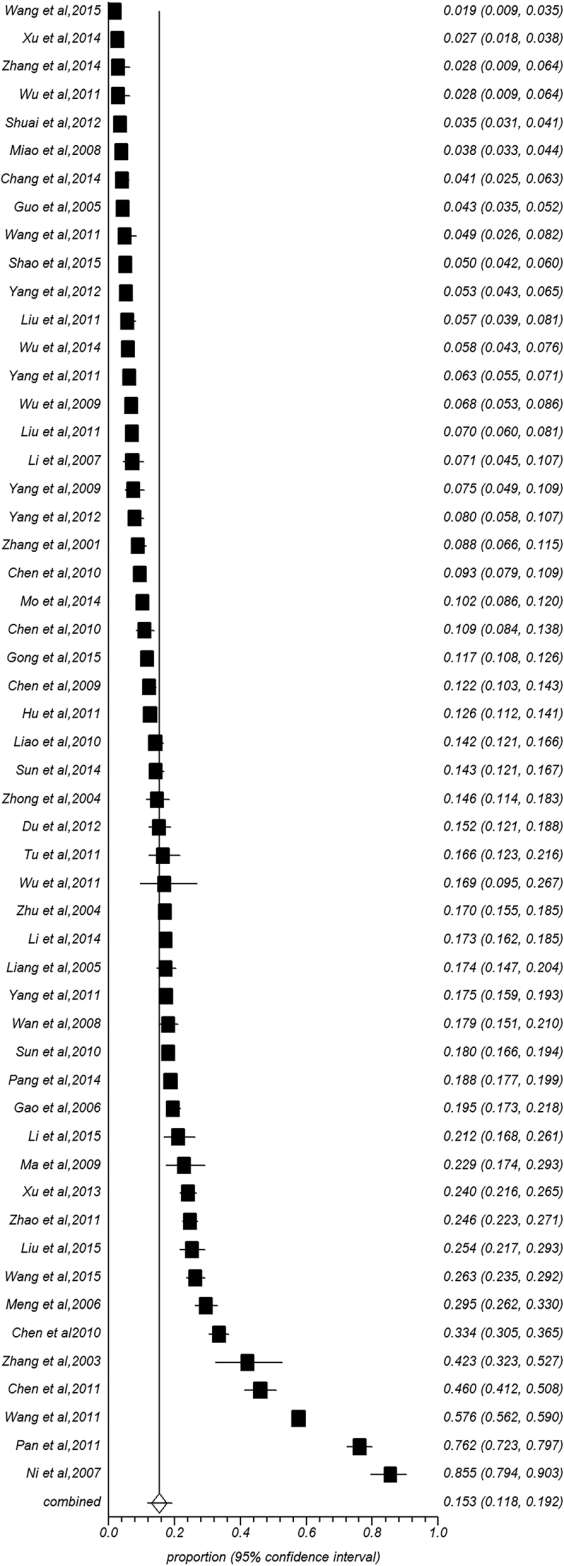
Forest plot of prevalence of osteoporosis for male

The prevalence rate of osteoporosis was significantly higher among females (25.41 %, 95 % CI: 21.54–29.49 %, Fig. 3) than males (15.33 %, 11.8–19.21 %, Fig. 4) (Table 1). In all age groups, the prevalence rates of osteoporosis increased with age. Specifically, the rate was the lowest (2.40 %) in the 15 to 30-year age group and the highest (56.10 %) in the 80 years and older age group in combined populations (6.49 % for 30- years, 8.88 % for 40- years, 19.57 % for 50- years, 35.10 % for 60- years, 43.38 % for 70- years). The prevalence rates among males (1.12 %, 3.43 %, 6.42 %, 11.64 %, 18.71 %, 28.6 %, 36.53 %) and females (2.18 %, 6.48 %, 10.10 %, 23.85 %, 45.77 %, 58.26 %, 68.45 %) were consistent with this overall trend. The overall prevalence in females was higher than the prevalence in males in each age group. Gender differences in osteoporosis prevalence in different regions and areas are shown in Tables 2 and 3. The prevalence rates in South China were higher among males than females (26.19 % vs. 17.95 %), while opposing results were identified in North China (12.22 % vs. 24.61 %). Similar phenomena did not appear in different regions. The prevalence rates were higher among females than males regardless of urban (22.96 % vs. 16.47) or rural (30.98 % vs. 12.15 %) residence. The gender difference was significant in rural but not in urban areas.

**Table 2:** Comparison of osteoporosis prevalence for sex in different regions and areas

Category	Subgroup	NO. of Studies	Prevalence *(95 % CI*)(%)	*N*	*I* ^*2*^ (%)	Publication bias	
						*P*(Begg)	*P*(Egger)
Southern	Female	29	17.95 [11.79–25.08]	39133	99.7	0.065	0.929
	Male	29	26.19 [20.46–32.35]	51945	99.6	0.246	0.145
Northern	Female	27	24.61 [19.32–30.31]	56445	99.5	0.348	0.366
	Male	23	12.22 [9.28–15.51]	36577	98.8	0.809	0.717
Urban	Female	38	22.96 [18.18–28.13]	64926	99.6	0.009	0.001
	Male	39	16.47 [11.91–21.61]	52689	99.6	0.007	0.007
Rural	Female	17	30.98 [23.98–38.46]	43263	99.6	0.205	0.046
	Male	14	12.15 [8.51–16.34]	23222	98.7	0.101	0.021

**Table 3:** Results of Meta-regression for Prevalence of osteoporosis

Covariate	Meta-regression coefficient	*95 %* Confidence interval	*P* value
Year of publication	−0.227	−0.086 to 0.002	0.061
Year of collecting	−0.239	−0.101 to −0.001	0.048
Female ration (%)	0.171	−0.198 to 1.099	0.170
Area (northern vs southern)	0.062	−0.217 to 0.364	0.614
Source (Hospital vs General)	0.276	0.055 to 0.672	0.022
Response rate (%)	0.245	0.000 to 0.059	0.051
Sample size, continuous	−0.320	−0.001 to 0.001	0.070
Region (Urban vs Rural)	0.064	−0.226 to 0.386	0.604
Criteria (WHO vs China)	−0.151	−0.479 to 0.110	0.215
Quality score	−0.012	−0.166 to 0.151	0.924
Age of onset	0.200	−0.002 to 0.021	0.099

#### Region and area

The prevalence rate of osteoporosis was slightly but not significantly lower in urban than in rural areas (20.87 % vs. 23.92 %; 95 %CI: 17.05–24.96 %). Similarly, the prevalence rate was slightly but not significantly higher in South China (23.92 %, 95 % CI: 18.29–28.44 %) than in North China (20.13 %, 15.62–25.06 %).

#### Diagnostic criteria and source of population

There were some differences (approximately 0.5 SD) in osteoporosis prevalence between studies using the WHO and Chinese criteria. The results showed that the pooled prevalence obtained from studies using the Chinese criterion was only slightly but not significantly higher than that obtained from studies using the WHO criteria (23.4 % vs. 20.35 %). The point estimate for osteoporosis prevalence obtained in the hospital setting was higher than that obtained from studies conducted in the general population (29.3 % vs. 19.7 %).

### Meta-regression analysis and publication bias

**Fig. 5:**
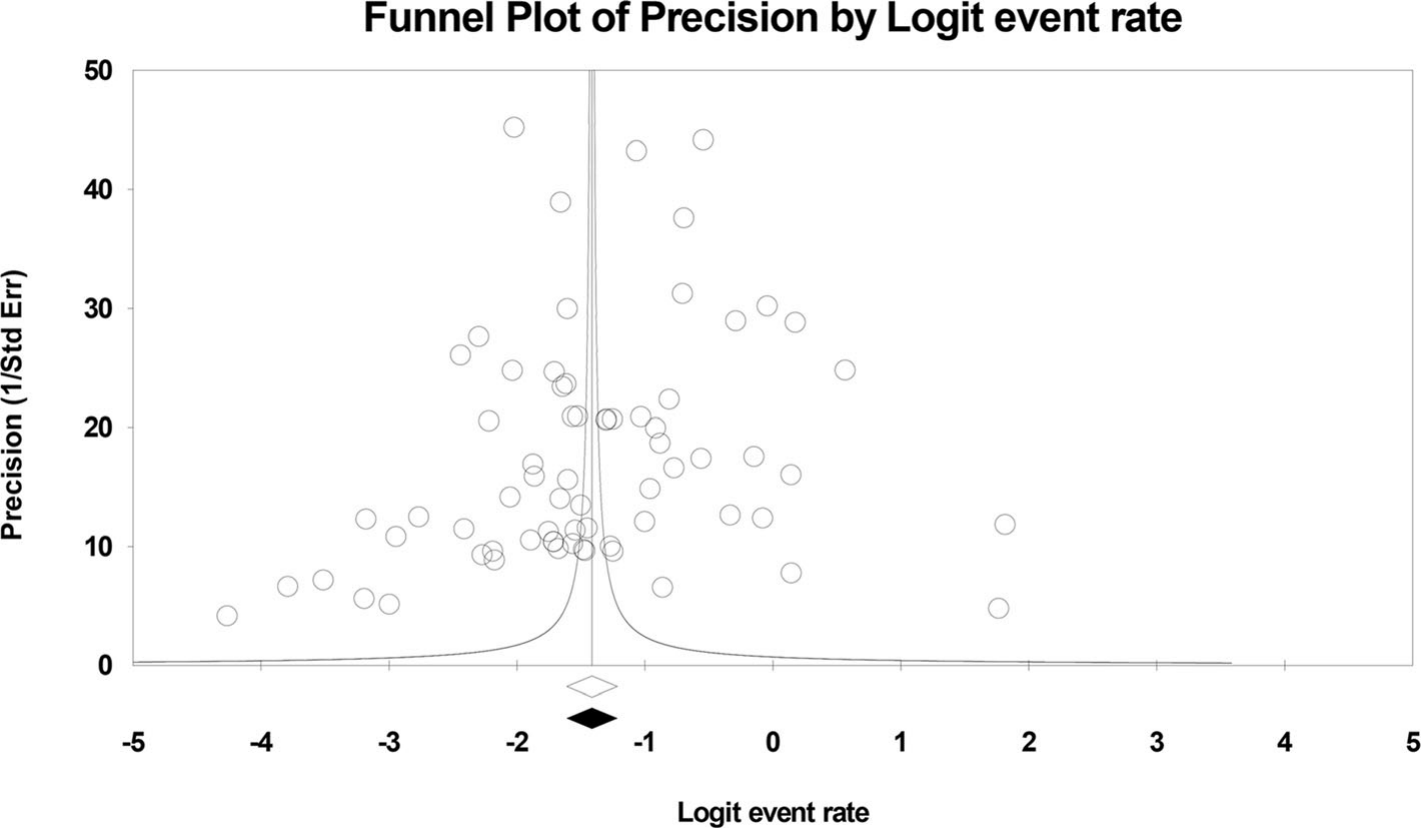
Funnel plot for publication bias

We observed that the heterogeneity across studies is particularly high when studies were evaluated overall. The *I*^*2*^ statistics ranged from 73.5 to 99.8 %. In the meta-regression analyses, overall prevalence estimates were not modified by the year of publication, female ratio (%), area, response rate, region, criteria, age at onset, or quality score. The results showed that year of data collection and study setting both significantly affected the estimation of point prevalence (*P* = 0.048, *P* = 0.022). Studies conducted in hospital populations were associated with an overestimated pooled prevalence of osteoporosis; however, this result does not fully explain the high level of heterogeneity observed. We generated a funnel plot including all studies; however, the funnel plot did not show evidence of asymmetry (Fig. 5). Analysis using Begg’s and Egg’s tests provided similar results (*P* = 0.297, *P* = 0.300). However, publication bias was still identified in some subgroups (Table 1).

## Discussion

This systematic review and meta-analysis was conducted to estimate the prevalence rates of osteoporosis among Chinese males and females, characterize the epidemiology of osteoporosis in China, and compare osteoporosis prevalence rates between the Chinese population and other populations. Several characteristics of osteoporosis in China were identified. First, the prevalence of osteoporosis obviously increased over the past decade (from a prevalence of 14.94 % before 2008 to 27.96 % in the period spanning 2012–2015). Second, the prevalence rate was higher in females than in males of the same age groups. Third, the prevalence was higher in rural areas than in urban areas and higher in South China than in North China. Fourth, at present, the pooled prevalence of osteoporosis in people above the age of 50 years is estimated to be more than twice the pooled prevalence in 2006 (34.65 % vs. 15.7 %) [18]. The prevalence of osteoporosis among the Chinese elderly population identified in this study was very high. Fifth, the application of different diagnostic criteria could have impacted prevalence estimation.

One of the main risk factors for osteoporosis in both males and females is ageing, which is a non-modifiable factor. The Korea National Health and Nutrition Examination Survey (KNHANES) showed an obvious age difference [19]. A large-scale survey in Austria indicated that the prevalence of osteoporosis increased with age [20]. A cohort study suggested that participants diagnosed at a young age had a higher prevalence of osteoporosis than those diagnosed at an older age (35 % vs. 10.0 %). It has been recognized that bone mineral density (BMD) decreases with age after reaching its optimal value. A clinical study including 17,083 subjects showed that the rates of bone mass reduction among women at 50–64 and ≥65 years of age were 31 and 62 %, respectively [21]. Our study was consistent with these studies. Sex is another non-modifiable factor. We found the prevalence rates of osteoporosis were remarkably higher among females than males in all age groups. According to a comprehensive review from Iraq, the prevalence of osteoporosis was 12 % among men, 3 % among premenopausal women, and 19 % among postmenopausal women, suggesting a significant gender difference [22]. Similar results have been reported in the United States (4.5 % vs. 15.4 %) [23]. One possible reason for the gender difference is that the oestrogen deficiency associated with menopause or oophorectomy may lead to a rapid BMD reduction in women, while in men, a decrease in testosterone levels may have a similar but less pronounced effect. An animal study showed that androgen receptor-mediated action was pivotal to bone maintenance in male mice, and oestrogens and androgens may regulate bone growth [24]. That could explain why postmenopausal women may be more prone to osteoporosis [25]. In this meta-analysis, we did not divide participants into premenopausal and postmenopausal subgroups. However, we still saw a significant increase in the prevalence of osteoporosis in females (from 10.1 to 23.85 %). Previous studies have also confirmed that women are at a higher risk of osteoporosis than men [26]. Another non-modifiable factor is race. We divided the studies into those conducted in South China and North China. The prevalence was higher in South China than North China. However, the prevalence rate among males in South China was higher than the rate among South Chinese females (26.19 % vs. 17.95 %), and opposing results were identified in North China (12.22 % vs. 24.61 %). These findings are in contrast with some previous studies. The prevalence of osteoporosis in North Iraq was found to be higher than that in South Iraq, and that regional difference was attributed to differences in vitamin D levels. As has been reported, vitamin D3 synthesis may not be sufficient to account for BMD reduction due to the lack of ultraviolet rays in high-latitude regions [27]. In addition to this factor, eating habits may account for some of the differences in osteoporosis prevalence in China. People in North China prefer cooked wheaten food, while people in South China live on rice and eat more aquatic products. Moreover, the climate is dry in North China and moist in South China, which also may account for the different results. However, the specific mechanism behind this difference is still unclear. Diet and lifestyle have been found to be associated with increased BMD [28]. Additionally, the prevalence of osteoporosis was slightly higher in rural than urban areas; this difference was probably due to disparities in health and medical resources. Osteoporosis is also a genetic disease. Those with a family history have a higher risk of osteoporosis; however, the heritability of BMD reduction has been found to vary widely from 25 to 80 %, and osteoporosis has been found to be associated with more than 30 genes [29].

Unlike advanced age, gender and race, eating habits are a modifiable factor. There are many other modifiable risk factors such as smoking, drinking, lower levels of physical activity, vitamin D deficiency, history of fracture, and calcium malabsorption [30]. In addition, China faces two major challenges now and in the future. First, China has the largest population in the world, which means an increased population with osteoporosis even if the incidence of osteoporosis is kept at the current level. Second, accelerated ageing in China necessitates the establishment of an all-functional social security system for the ageing population. As is well known, people aged 50 and years and above are at an increased risk of osteoporosis. As reported in 2010, there were 111 million (8.2 % of China’s population) elderly Chinese individuals (aged 65+ years), of which 19.3 million were in the oldest age group (aged 80+). The elderly population aged 65+ years is estimated to increase immensely, reaching 400 million by 2050 [30]. Though the one-child policy was further loosened in November 2013 after the Third Plenary Session of the 18th CPC Central Committee, its current form stipulates that couples are allowed to have two children if one of them is an only child. However, this change might result in unintended consequences [31].

Given the current epidemiology of osteoporosis in China, preventive and control measures are needed to increase the awareness of citizens regarding this condition through three-step prevention programmes. Maximization of bone mass is the key to preventing osteoporosis. According to the present results, osteoporosis is an age-related disease. Calcium and vitamin D supplementation are needed, especially in females and people aged 50 years and above. People in the hospital setting were found to have a higher prevalence of osteoporosis; however, samples obtained from the general population may have underestimated the prevalence of osteoporosis, and more effective screening methods are needed.

This study has some limitations. First, more females than males were included, which may have resulted in an overestimate of the prevalence of osteoporosis, as it occurs more frequently among females. Second, the onset ages in the included studies were categorized differently, which we believe could affect the results in some subgroups since the point prevalence of osteoporosis was found to increase with increased age. Third, heterogeneity was relatively high in all analyses. Although we identified one factor affecting these results, the degree of explainability was very limited. Nevertheless, the main strengths of this study were that most of the included studies had large sample sizes. Two investigators independently extracted data and reviewed the articles to obtain data accurately. We report the results in accordance with the PRISMA statement.

## Conclusions

The prevalence of osteoporosis in China has increased over the past 12 years, affecting more than one-third of the people aged 50 years and older. The prevalence of osteoporosis was found to increase with age and was higher in females than males. Prevention and control measures have become more important given the increase in the prevalence of osteoporosis.

## Additional files

Additional file 1: Table S1. China and international osteoporosis diagnosis criteria. (DOC 30 kb) Additional file 2: PRISMA 2009 Checklist. (DOC 64 kb) Additional file 3: Studies included in the meta-analysis. (DOC 47 kb) Additional file 4: Characteristic of Studies on the Prevalence of osteoporosis. (DOC 256 kb)

## Abbreviations

IPV: Intimate partner violence
