# Supplementary material for: Prevalence of osteoporosis in China: a meta-analysis and systematic review
Source: BMC Public Health. 2016 Oct 3;16:1039. doi: 10.1186/s12889-016-3712-7 (PMC5048652; doi:10.1186/s12889-016-3712-7)
Supplement: Additional file 1: Table S1. — China and international osteoporosis diagnosis criteria. (DOC 30 kb) [file 12889_2016_3712_MOESM1_ESM.doc]

**Supplementary materials 1**

Table S1 China and international osteoporosis diagnosis criteria

| Classification of diagnosis criteria | WHO  (standard deviation) | OCCGS ( [standard](javascript:void(0);) [deviation](javascript:void(0);)) | OCCGS  (bone mass reduction percent,%) |
| --- | --- | --- | --- |
| Normal | ≥-1.0 SD | ±1.0 SD | ±12% (include 12%) |
| Osteopenia | -1.0 SD to -2.5 SD | -1.0 to -2.0 SD | -13% to -24% (include 24%) |
| Osteoporosis | ≤-2.5 SD | ≤SD | ≥25% |
| Severe osteoporosis | ≤-2.5 SD with one or more fracture | ≤-2.0 SD with one or more fracture | ≥25% with one or more fracture, or ≥37% without fracture |

WHO: Guideline for preclinical evaluation and clinical trials in osteoporosis, 1998, Geneva; WHO: WHO scientific group on the assessment of osteoporosis at primary health care level, summary meeting report, Brussels, Belgium,5-7 May 2004.

Osteoporosis committee of China gerontological society(OCCGS).
