# Supplementary material for: Prevalence of osteoporosis in China: a meta-analysis and systematic review
Source: BMC Public Health. 2016 Oct 3;16:1039. doi: 10.1186/s12889-016-3712-7 (PMC5048652; doi:10.1186/s12889-016-3712-7)
Supplement: Additional file 3: — Studies included in the meta-analysis. (DOC 47 kb) [file 12889_2016_3712_MOESM3_ESM.doc]

**Supplementary 3 Studies included in the meta-analysis**

1. Chuang Xu，Hui Jin，Zhende Jiang, Jincheng Wang. Comparison of epidemiology study of osteoporosis among 210 females. Modern Med. 2015; 03:496-498. (in Chinese)

2. Li C, LV WH, Wang TT, Xing YR, Zhang LX, Liu XG, Wang ZF, Zheng LL. The study of prevalence of osteoporosis in 1088 medical workers. Chin J Osteoporos. 2015; 10:1217-1220.

3.Pan CH, Tan QY, Li WF, Xu Y, Xia F. Analysis of bone density and osteoporosis in 1157 old population in hospital. Cotemporary Med. 2011; 15:81-82.

4. Wu K, Zhou HQ, Wang GC, Wu HD, Limn B, Ma L, Zhao MJ, Wang LY, Lu X, Zhang L, Zu N. An assessment of osteoporosis awareness in older patients of out-patient clinic. Chin J Osteoporos. 2011; 6:503-531.

5. Kang S, Zhu XY. Comparison of osteoporosis and related behavior between hospital and community. Chin J Gerontol. 2008; 12:2356-2358.

6.Chen T, Jiang BQ, Xue JL, Zhong PH, An JJ, Song XY, Yang J, Sun HL, Fan Y. The investigation of prevalence rate of osteoporosis among teachers in Chongqing urban district. Parentera& Enteral Nutrition. 2010; 5:288-290.

7.Gong XH, You L, Ma JH. Epidemiological analysis of relationship between blood lipids and osteoporosis. Investigation and Research. 2015; 12:113-114

8.Liu P, Hu P, Xing FX, Liu XM, Huang HM, Huang Z, Wang Y. Prevalence of osteoporosis and analysis of bone density among middle and older population. Chin J Gerontol. 2013； 33:5139-5140.

9.Chenl QS, Lou HL, Peng C, Wang HW, Zhu GH, Qiu HC. Analysis of bone density and osteoporosis among middle-aged people. Guangdong Med. 2011; 5:620-622.

10. Guo LY, Zhou LF, Yang J, Gao ES. Investigation on knowledge and prevalence of osteoporosis in mid-aged and elderly population in a community of Shanghai. Chin J Public Health. 2015; 4:470-472.

11.Zhu ZX, Zhang JH, Gu XX, Dai YH. Measurement of bone mineral density with Dove3000 osteoanalyzer and prevalence of osteoporosis in 6330 residents in north Zhejiang. Chin J Osteoporosis. 2004; 2: 191-192.

12. Zhang FY, Deng ZS, Wu F. Effects of age and sex on primary osteoporosis. Chin J Modern Med. 2003; 4:48-52.

13. Sun TM, Qi PY, Yin XH, Lin M. Investigation of osteoporosis in 1932 middle-aged and aged women in Yuxi. Chin J Osteoporos. 2010; 6: 424-425.

14. Sun TM, Qi PY, Yin XH, Lin M. Investigation of osteoporosis in 1932 middle-aged and aged men in Yuxi. Chin J Osteoporos. 2010; 4: 292-294.

15. Ma XJ, Kong SL, Meng XJ, Dong JY, Zhang J, Zhang LG, Zhang QQ, Shi NN. The factors associated with osteoporosis among adult women. Chin J Pract Inter Med. 2014; 1:78-81.

16.Yang WD, Yang J, Yu B, Liu YP. Factors and prevalence of osteoporosis among old population in Yichang. Med Frontier. 2012; 5:75-76.

17. Du XM, Li ZQ, Xu ZY. Investigation of bone mineral and osteoporosis prevalence among the elderly in Yanji Community. Control and Prev Dise. 2012; 14:41-42.

18.Chang GQ, Lou PA, Chen PP, Qiao CC, Li RG, Jiang YC, Zong H, Zhang YQ, Pann XQ, Lin P. Survey of incidence and awareness of osteoporosis of residents aged 40-60 years in Jiawang District of Xuzhou City. Chin J School Doctor. 2014; 8:583-586.

19.Xia YJ, Han J, Wang SH. Analysis of osteoporosis among postmenopausal women in Xinjiang. Chin Prac Med. 2011; 6: 267-268.

20. Miao Y, Peng B, Qiao HF. Survey of prevalence of primary osteoporosis in Kelamyi area of Xinjiang. Chin J Osteoporos. 2010; 8:598-586.

21.Jiang BX, Wang Y, Ha FL, Wang J. analysis of patient with osteoporosis in Dushanzi area. Chin J Osteoporos. 2012; 4:342-343.

22. Zeng YH, Zhang B, Qiu H, Pan MM, Shi J, Liu F. Prevalence of osteoporosis among general population in Xian City. Chin J Clinicians. 2012; 15: 4449-4450.

23. Li ZQ, Wei RQ, Wang LM, Li XG, Zhuang QJ, Ni Y. Measurement of bone mineral density and analysis of osteoporosis incidence in Xian area. Med J NDFNC. 2007; 4: 275-276.

24. Hu YL, Tao Y. Investigation of prevalence of osteoporosis and risk factors among middle-aged elderly in Wuhan City. J Bethune Milit Med College. 2011; 2: 99-100.

25. Xu LJ, Guo GB, Tang SQ. The relationship between body mass indes and morbidity osteopenia of osteoporosis. Chin J Med Guide. 2013; 11:1817-1818.

26. Zhao XF. Survey on prevalence of osteoporosis in employees of Suzhou Health and Paramedical College. J Environ Occup Med. 2011; 7: 442-444.

27. Yang MW, Wang ST, Wen LL, Chen X. Analysis of bone mineral density in normal people in Shenyang and Yingkou of China. Chin J Osteoporos. 2009; 3: 179-182.

28.Li SY, Deng AP, Li XW. Measurement of bone mineral density and analysis of osteoporosis incidence in Shenzhen City. Chin J CT MRI. 2012; 43:118-120.

29. Mo QiH, Yu HZ, <o QW, Lin LY, Ma T, Miao ZC. Investigation of osteoporosis among population aged 50 in a community of Shenzhen City. 2013; 7:1429-1430.

30. Wang HM. Investigation of bone mineral density and osteoporosis prevalence in 8345 civil services from Shenzhen. Chin Modern Doct. 2011; 20: 11—30.

31. Zhong ZR, Liang XP, Zhou WY, Wen JL, Zhou YY. Evaluation of bone mineral and osteoporosis prevalence in different age group of Shenzhen. Guangdong Med J. 2004; 10:1186-1187.

32. Wu FT, Hu CL, Fang YF, Wan YH, Tao YH, Zhang FK, Chen WS. Prevalence and influencing Factors of osteoporosis among mid-aged and elderly community residents. Chin General Prac. 2011;5B；11540-11542.

33. Ma JL, He MH, Gu ZN, Huang SX< Guo HY, Yang XD, Jiang WZ, Sun YH, Hou QW, Shi Y, Shi L, Zhang Y, Ji GC. Epidemiolgical investigation of osteoporosis in aged people in Beicai Town of Shanghai. Chin J Osteoporos. 2009; 12: 932-935.

34. Wang YM, Yang M, Lv ZL, Qian W, Huang H. Survey of the osteoporosis morbidity rate and effective factor of the mid and elderly population in some community of Shanghai suburbs. Community Healthcar. 2011; 6: 408-411.

35. Yang CY, Qiu QF, Zhai XJ, Cong ZL, Wang HL, Li SL. Investigation of the correlation between bone mineral density and bone metabolism index in health adults in Jiaodong Peninsula. Chin J Osteoporos. 2011; 5: 431-439.

36. Yi XY.Epidemiology survey about women op over 35 years old of Qinghai. J Qinghai Med College. 2004; 3: 164-167.

37. Pang Z, Liu N, Zhang Y, Chen L. Analysis of bone mineral density and osteoporosis prevalence in 6390 population of Panjin City. Chin Prac Med. 2014; 3: 89-90.

38. Ni Z, Jiang S. Investigation of bone mass density and effects of factors on osteoporosis in healthy man in Nigbo. Chin J Osteoporos. 2007; 9: 627-630.

39. Wang CC, Honh Q, Wang ZY, Yang HF, Chen XP, Qi SX, Zhou HR, Xu F. Analysis on common chronic diseases among osteoporosis patients and high risk population in Nanjing City. Occup and Health. 2015; 20: 2845-2847.

40.Lan LF, Hu AZ. Fang C. Prevalence of osteoporosis in community females aged over forty and its influencing factors. J Nurs(China). 2013; 4B:33-36.

41. TP, Deng B, Xu DB, Wang Y, Duan P, Ding X. Study on the correlation between the alteration of bone mass and bone metabolism marker in residents aged more than 20 years old in Nanchang. Chin J Osteoporos. 2011; 3: 249-252.

42. Zhang FK, Wang YF, Liu GY, Fang YF, Wu FT, Hu CL. Influence factors of osteoporosis among middle-aged and older population in communities. Anhui Med and Pharm J. 2014; 18:637-639.

43. Zhao J, Zhang YY, Zhang B. Study on the correlation between osteoporotic fractures and bone mineral density in the proximal femur in the elderly in Luohe. Chin JOsteoporos. 2014; 10: 1216-1221.

44. Meng YJ, Wei JY, Long LY, He ZH, Wu DM. Investigation of osteoporosis prevalence among the elderly in Liuzhou City. Qiqihaer Med College. 2006; 15: 1851.

45.Miao YQ. Zhao MS. The investigation of epidemiology status of osteoporosis and bone mineral density among common health people in Lanzhou area.Chin Health Industry. 2015; 7:178-180.

46. Zhang H. Zhou HQ, Du FH, Guo SL, Zhang A. Bone mineral density of adult people in Lanzhou area. Chin J Osteoporos. 2003; 4: 335-336.

47. Shen ZG, Wu P, Xiao N, Chen WH. Investigation the usage of anti-osteoporosis drugs in people over the age of 60 JiaXin Road communities. Geriatric Health Care. 2013; 6: 391-394.

48.Li GC, Zhou MY. Li CM. Investigation and analysis of incidence of osteoporosis for 9735 cases of bone mineral density population in Jlin city region. Chin J Med Frontier. 2014; 11: 90-93.

49. Wu ZQ, Wu YF, Hu BJ, Gao DW, Yuan YX, Xu L. Investigation of primary osteoporosis and risk factors in 1263 middle-aged and elderly in the area of Guangdong. J New Chin Med. 2013; 10: 51- 53.

50. Sun Y, Zhou XQ, Liao Y, Wang SX, Zhang Y. The study of the correlation between obesity and primary osteoporosis in community. Chin J Osteoporos. 2014; 4: 439-442.

51.Shao ZD, Li TL, Yuan SQ, Chen SH, Zhu XP, Wang Y, Li XH, Zhou CX, Wang DJ, Qin TB, Me TC, Wu SL. Characterization of bone mineral density and incidence of osteoporosis among coal miners in north China. Modern Preve Med. 2015; 6:991-993.

52. Liu HW, Chen JY, Zhang GH, Luo ZR, Chen XA, Lin PY. A cross-sectional survey of bone mass and a analysis of risk factors for osteoporosis in 4000 health adults in Shaoyang region of Hunan Province. Chin J Osteoporos. 2011; 12: 1087-1091.

53. Chen XP, Wang G, Wan C, Yin J, Zhu XH. Study bone mineral density of normal bodies in Shiyan. Chin J Osteoporos. 2010; 3: 210-212.

54. Chen WG, Zheng LW, Guo XH. Investigation of bone mineral density among middle-aged and elderly in Huhaohaote. Chin J Osteoporo. 2009; 29:764.

55. Mo JX, Meng WW, Ye H, Mo CR, Tang SY, Mo JF. Analysis of the survey of the prevalence osteoporosis of 2451 health people in Guibei area. J Clin Res. 2014; 1; 137-138.

56. Liu L, Zhang Q, Peng NC. Investigation on osteoporosis status in health adults in Guiyang of China.Chin J Osteoporosis and bone miner Res. 2011; 2: 108-112.

57. Liang WJ, Huang XF, Wei RY, Luo ZW, Ma YJ, Mo Jl. Research of osteoporosis mobidity on female Chuang in urban and rural areas of Baise Guangxi. Anat Res. 2005; 3: 188-189.

58. Gao JH, Zheng JJ, Zhang RP, Gu H, Zhang MG, Suo P. Bone mineral density measurement and prevalence of osteoporosis in 2454 citizen in Jiangmen District of Guangdong Province. 2006; 5: 395-397.

59. Xu L, Wang J, Zhu XH, Cui SW, Yang JJ. An analysis of detection of bone mineral density and prevalence of osteoporosis in out-patient department of osteoporosis. Jiangsu Med J. 2014; 20:2422-2424.

60. Wu LB, Liu G. Li FY. Pei ZJ. Chen Y Wang WM. Investigation and analysis of bone mineral density with normal bodies in the northwest of Hubei Province. Chin J Osteoporos.2009; 15:432-434/.

61.Chen HY, Pu XJ, Yu N. Screening osteoporosis in healthy adult of luogan district Guangzhou city using quantitative ultrasound technique. J Hebei Med Univ. 2010; 12; 1474-1476.

62. Yang SQ, Ma BC, Liu XY, Men BL. Prevalence of osteoporosis among 4633 adults in Chifeng region. Inner Mongolia Med J. 2012; 7； 834-836.

63. Shuai P, Cheng YF, Liu YP, Wang L, Ren JJ, Gong LR, Zheng XX, Wu YP, Xiao X. The investigation of epidemiology status of osteoporosis and bone mineral density among common health people in Chengdu area. Sichuan Med J. 2012; 9: 1680-1682.

64.Wang P, Hao GX, Huang CY, Zhou M. Analysis of osteoporosis among different age group. Chin Clinica J. 2015; supply:89-91.

65. Wan ZH. Analysis of bone mineral density and influencing factos among retired teachers in East district of Beijing. Chin J Sch Health. 2008; 11； 1064-1065.

66. WangJY, Gong HF, Ren LX, Feng SX, Lin L. Epidemiology Investigation of osteoporosis in the Chaoyang District of Beijing. Chin J Prim Med Pham; 2012; 18: 2770-2771.

67. Wang JH. Zhang ZH Zhong P, Wang QL, Shirly XB Lauai AD. Investigation and analysis the morbidity of osteoporosis of Beijing women. Aerospace Med. 2008; 2； 85- 86.

68. Yang HB, Qian ZF, Li Q, Guo Ml, Wang C, He XY, Liu ZH. Investigation of bone mineral density on the distal forearm and analysis of prevalence of osteoporosis in 19609 health people in miyun county of Beijing. Chin J Osteoporos. 2011; 1: 51-55.

69. Li S, He H, Ding M, He C. The correlation of osteoporosis to clinical features: a study of 4382 Female Cases of a Hospital Cohort with musculoskeletal symptoms in Southwest China. BMC Musculoskeletal Disorders. 2010; 11:183.
