# Supplementary material for: Prevalence of osteoporosis in China: a meta-analysis and systematic review
Source: BMC Public Health. 2016 Oct 3;16:1039. doi: 10.1186/s12889-016-3712-7 (PMC5048652; doi:10.1186/s12889-016-3712-7)
Supplement: Additional file 4: — Characteristic of Studies on the Prevalence of osteoporosis. (DOC 256 kb) [file 12889_2016_3712_MOESM4_ESM.doc]

**Supplementary 4 Characteristic of Studies on the Prevalence of osteoporosis**

| **NO.** | **First author** | **Publication**  **Year** | **Screening**  **Year** | **Province** | **Study design** | **Area** | **Region** | **Minimum age** | **Sex**  **(M)** | **Case**  **(n)** | **Sample**  **size** | **Prevalence (%)** |
| --- | --- | --- | --- | --- | --- | --- | --- | --- | --- | --- | --- | --- |
| 1 | Xu et al | 2015 | 2015 | Liaoning | Cross-sectional | Northern | Mixed | 20 | 1.00 | 60 | 201 | 0.30 |
| 2 | Li et al | 2015 | 2014 | Henan | Cross-sectional | Northern | Urban | 40 | 0.71 | 303 | 1088 | 0.28 |
| 3 | Pan et al | 2011 | 2010 | Shanghai | Cross-sectional | Southern | Urban | 45 | 0.54 | 996 | 1157 | 0.86 |
| 4 | Wu et al | 2011 | 2010 | Beijing | Cross-sectional | Northern | Urban | 45 | 0.86 | 115 | 616 | 0.19 |
| 5 | Kang et al | 2008 | 2007 | Heilongjiang | Cross-sectional | Northern | Urban | 50 | 0.72 | 86 | 838 | 0.10 |
| 6 | Chen et al | 2010 | 2006 | Chongqing | Cross-sectional | Southern | Urban | 40 | 0.52 | 1462 | 3401 | 0.43 |
| 7 | Gong et al. | 2015 | 2013 | Guangdong | Cross-sectional | Southern | Urban | 45 | 0.56 | 1799 | 11180 | 0.16 |
| 8 | Liu et al | 2015 | 2011 | Beijing | Cross-sectional | Northern | Urban | 45 | 0.60 | 473 | 1296 | 0.36 |
| 9 | Chen et al | 2011 | 2010 | Guangdong | Cross-sectional | Southern | Urban | 40 | 0.55 | 550 | 1024 | 0.54 |
| 10 | Guo et al | 2005 | 2005 | Shanghai | Cross-sectional | Southern | Urban | 50 | 0.55 | 465 | 4708 | 0.10 |
| 11 | Zhu et al | 2004 | 2005 | Zhejiang | Cross-sectional | Southern | Rural | 20 | 0.62 | 2117 | 6330 | 0.33 |
| 12 | Zhang et al | 2003 | 2003 | Hunan | Cross-sectional | Southern | Rural | 19 | 0.58 | 128 | 238 | 0.54 |
| 13 | Sun et al | 2010 | 2010 | Yunnan | Cross-sectional | Southern | Urban | 40 | 1.00 | 555 | 1932 | 0.29 |
| 14 | Sun et al | 2010 | 2009 | Yunan | Cross-sectional | Northern | Urban | 20 | 0.00 | 531 | 2953 | 0.18 |
| 15 | Ma et al | 2014 | 2013 | Shangdong | Cross-sectional | Northern | Urban | 18 | 1.00 | 32 | 807 | 0.04 |
| 16 | Yang et al | 2012 | 2011 | Hubei | Cross-sectional | Northern | Urban | 60 | 0.58 | 220 | 1200 | 0.18 |
| 17 | Du et al | 2012 | 2012 | Shanghai | Cross-sectional | Southern | Urban | 50 | 0.68 | 232 | 1447 | 0.16 |
| 18 | Chang et al | 2014 | 2013 | Jiangsu | Cross-sectional | Southern | Urban | 40 | 0.75 | 44 | 1979 | 0.02 |
| 19 | Xia et al | 2011 | 2011 | Xinjiang | Cross-sectional | Northern | Rural | 40 | 1.00 | 198 | 733 | 0.27 |
| 20 | Liao et al | 2010 | 2010 | Xinjiang | Cross-sectional | Northern | Urban | 40 | 0.62 | 542 | 2516 | 0.22 |
| 21 | Jiang et al | 2012 | 2008 | Xinjiang | Cross-sectional | Northern | Rural | 45 | … | 147 | 990 | 0.15 |
| 22 | Zeng et al | 2012 | 2012 | Shangxi | Cross-sectional | Northern | Urban | 20 | 0.50 | 27 | 564 | 0.05 |
| 23 | Li et al | 2007 | 2007 | Shanxi | Cross-sectional | Northern | Urban | 20 | 0.44 | 117 | 522 | 0.22 |
| 24 | Hu et al | 2011 | 2010 | Bubei | Cross-sectional | Southern | Urban | 40 | 0.49 | 651 | 4000 | 0.16 |
| 25 | Xu et al | 2013 | 2012 | Hubei | Cross-sectional | Southern | Urban | 21 | 0.28 | 491 | 1668 | 0.29 |
| 26 | Zhao et al | 2011 | 2009 | Jiangsu | Cross-sectional | Northern | Rural | 20 | 0.44 | 589 | 2230 | 0.26 |
| 27 | Yang et al | 2009 | 2008 | Jilin | Cross-sectional | Southern | Urban | 21 | 0.44 | 113 | 600 | 0.19 |
| 28 | Li et al | 2012 | 2012 | Guangdong | Cross-sectional | Southern | Urban | 21 | … | 113 | 713 | 0.16 |
| 29 | Mo et al | 2013 | 2012 | Guangdong | Cross-sectional | Southern | Urban | 53 | 0.64 | 126 | 956 | 0.13 |
| 30 | Wang et al | 2011 | 2010 | Guangong | Cross-sectional | Southern | Urban | 20 | 0.39 | 3080 | 8345 | 0.37 |
| 31 | Zhong et al | 2004 | 2003 | Guangdong | Cross-sectional | Southern | Urban | 20 | 0.50 | 155 | 876 | 0.18 |
| 32 | Wu et al | 2011 | 2010 | Anhui | Cross-sectional | Northern | Urban | 64 | 0.78 | 126 | 820 | 0.15 |
| 33 | Ma et al | 2009 | 2008 | Shanghai | Cross-sectional | Southern | Rural | 60 | 0.66 | 293 | 608 | 0.48 |
| 34 | Wang et al | 2011 | 2009 | Shanghai | Cross-sectional | Southern | Rural | 56 | 0.69 | 163 | 853 | 0.19 |
| 35 | Yang et al | 2011 | 2010 | Shandong | Cross-sectional | Northern | Rural | 52 | 0.49 | 156 | 3879 | 0.04 |
| 36 | Yi et al | 2004 | 2004 | Qinghai | Cross-sectional | Northern | Rural | 35 | 1.00 | 1783 | 3632 | 0.49 |
| 37 | Pang et al | 2014 | 2013 | Liaoning | Cross-sectional | Northern | Urban | 30 | 0.21 | 1074 | 6390 | 0.17 |
| 38 | Ni et al | 2007 | 2007 | Zhejiang | Cross-sectional | Southern | Urban | 30 | 0.00 | 153 | 179 | 0.85 |
| 39 | Wang et al | 2015 | 2013 | Jiangsu | Cross-sectional | Southern | Urban | 40 | 0.71 | 52 | 1784 | 0.03 |
| 40 | Lan et al | 2013 | 2011 | Jiangxi | Cross-sectional | Southern | Urban | 40 | 1.00 | 690 | 5936 | 0.12 |
| 41 | Tu et al | 2011 | 2011 | Jiangxi | Cross-sectional | Southern | Urban | 20 | 0.54 | 127 | 576 | 0.22 |
| 42 | Zhang et al | 2014 | 2013 | Anhui | Cross-sectional | Northern | Urban | 63 | 0.78 | 126 | 820 | 0.15 |
| 43 | Zhao et al | 2014 | 2010 | Henan | Cross-sectional | Northern | Urban | 45 | 0.30 | 272 | 649 | 0.42 |
| 44 | Meng et al | 2006 | 2005 | Guangxi | Cross-sectional | Southern | Urban | 40 | 418.00 | 572 | 1230 | 0.47 |
| 45 | Miao et al | 2008 | 2007 | Gansu | Cross-sectional | Northern | Urban | 21 | 0.47 | 836 | 9100 | 0.09 |
| 46 | Zhang et al | 2006 | 2006 | Gansu | Cross-sectional | Northern | Rural | 20 | 0.44 | 101 | 996 | 0.10 |
| 47 | Shen et al | 2013 | 2012 | Shanghai | Cross-sectional | Southern | Urban | 60 | … | 94 | 999 | 0.09 |
| 48 | Li et al | 2014 | 2013 | Liaoning | Cross-sectional | Northern | Urban | 20 | 0.56 | 2509 | 9735 | 0.26 |
| 49 | Wu et al | 2014 | 2013 | Guangdong | Cross-sectional | Southern | Rural | 45 | 0.67 | 401 | 1263 | 0.32 |
| 50 | Sun et al | 2014 | 2014 | Xinjiang | Cross-sectional | Northern | Urban | 21 | 0.62 | 537 | 2498 | 0.21 |
| 51 | Shao et al | 2015 | 2012 | Hebei | Cross-sectional | Northern | Urban | 23 | 0.00 | 122 | 2423 | 0.05 |
| 52 | Liu et al | 2011 | 2010 | Hunan | Cross-sectional | Southern | Rural | 25 | 0.44 | 667 | 4000 | 0.17 |
| 53 | Chen et al | 2010 | 2009 | Hubei | Cross-sectional | Southern | Rural | 15 | 0.51 | 526 | 3026 | 0.17 |
| 54 | Chen et al | 2009 | 2008 | Neimeng | Cross-sectional | Northern | Urban | 30 | 0.48 | 290 | 2147 | 0.14 |
| 55 | Mo et al | 2014 | 2012 | Guangxi | Cross-sectional | Southern | Urban | 20 | 0.47 | 328 | 2451 | 0.13 |
| 56 | Liu et al | 2011 | 2010 | Guizhou | Cross-sectional | Southern | Urban | 20 | 0.59 | 17 | 1217 | 0.01 |
| 57 | Liang et al | 2005 | 2004 | Guangxi | Cross-sectional | Southern | Rural | 25 | 1.00 | 125 | 718 | 0.17 |
| 58 | Gao et al | 2006 | 2005 | Guangdong | Cross-sectional | Southern | Urban | 20 | 0.50 | 548 | 2454 | 0.22 |
| 59 | Xu et al | 2014 | 2013 | Jiangsu | Cross-sectional | Southern | Urban | 20 | 0.58 | 164 | 2756 | 0.06 |
| 60 | Wu et al | 2009 | 2008 | Hubei | Cross-sectional | Northern | Urban | 15 | 0.51 | 224 | 1957 | 0.11 |
| 61 | Chen et al | 2010 | 2009 | Zhejiang | Cross-sectional | Southern | Urban | 20 | 0.69 | 142 | 1717 | 0.08 |
| 62 | Yang et al | 2012 | 2011 | Neimeng | Cross-sectional | Northern | Rural | 31 | 0.62 | 715 | 4633 | 0.15 |
| 63 | Shuai et al | 2012 | 2011 | Sichuan | Cross-sectional | Southern | Rural | 20 | 0.41 | 735 | 9111 | 0.08 |
| 64 | Wang et al | 2015 | 2013 | Shangxi | Cross-sectional | Northern | Urban | 21 | 0.58 | 719 | 2324 | 0.31 |
| 65 | Wan et al | 2008 | 2008 | Beijing | Cross-sectional | Northern | Urban | 50 | 0.80 | 1817 | 3331 | 0.55 |
| 66 | Wang et al | 2012 | 2011 | Beijing | Cross-sectional | Northern | Rural | 50 | 1.00 | 1695 | 2651 | 0.64 |
| 67 | Wang et al | 2008 | 2007 | Beijing | Cross-sectional | Northern | Rural | 41 | 1.00 | 291 | 1726 | 0.17 |
| 68 | Yang et al | 2011 | 2010 | Beijing | Cross-sectional | Northern | Rural | 20 | 0.81 | 2306 | 19609 | 0.12 |
| 69 | Li et al | 2010 | 2008 | Sichuan | Cohort study | Southern | Urban | 50 | 1.00 | 1455 | 4382 | 0.33 |

**Table S2 Characteristic of Studies on the Prevalence of osteoporosis (Continued Table S1)**

| **NO.** | **First author** | **Response rate (%)** | **Sample selection method** | **Sample source** | **Diagnostic criteria** | **Equipment of measurement** | **Quality**  **score** |
| --- | --- | --- | --- | --- | --- | --- | --- |
| 1 | Xu et al | 100.0 | Clustered randomized sampling | Hospital-based | WHO | SONOST3000 | 4 |
| 2 | Li et al | 100.0 | Clustered randomized sampling | General population | WHO | USA Hologic | 8 |
| 3 | Pan et al | 100.0 | Whole sample | Hospital-based | WHO | USA Norland | 8 |
| 4 | Wu et al | 100.0 | Whole sample | Hospital-based | China | USA Lunar enCORE2004 | 8 |
| 5 | Kang et al | 88.2 | Whole sample | General population | WHO | N/A | 7 |
| 6 | Chen et al | 100.0 | Clustered randomized sampling | General population | China | USALunar enCORE2004 | 6 |
| 7 | Gong et al. | 100.0 | Clustered randomized sampling | General population | WHO | France Medilnk | 8 |
| 8 | Liu et al | 100.0 | Whole sample | Hospital-based | WHO | Korea osteosys | 7 |
| 9 | Chen et al | 100.0 | Whole sample | Hospital-based | WHO | France Lexxos | 8 |
| 10 | Guo et al | 97.6 | Clustered randomized sampling | General population | China | N/A | 7 |
| 11 | Zhu et al | 100.0 | Whole sample | Hospital-based | China | USA Dove3000 | 9 |
| 12 | Zhang et al | 100.0 | Clustered randomized sampling | General population | WHO | Scanner SN | 5 |
| 13 | Sun et al | 100.0 | Clustered randomized sampling | General population | WHO | Japan DCS-600EX | 9 |
| 14 | Sun et al | 100.0 | Whole sample | Hospital-based | China | Japan DCS-600EX | 9 |
| 15 | Ma et al | 100.0 | Whole sample | Hospital-based | China | USA Lunar | 8 |
| 16 | Yang et al | 100.0 | Whole sample | Hospital-based | China | N/A | 7 |
| 17 | Du et al | 100.0 | Clustered randomized sampling | General population | WHO | Shanghai CM-200 | 9 |
| 18 | Chang et al | 100.0 | Clustered randomized sampling | General population | WHO | BMD1000A | 6 |
| 19 | Xia et al | 100.0 | Whole sample | General population | WHO | USALunar enCORE | 6 |
| 20 | Liao et al | 100.0 | Whole sample | General population | WHO | HOLOGIC | 9 |
| 21 | Jiang et al | 100.0 | Whole sample | General population | WHO | USA GE | 8 |
| 22 | Zeng et al | 100.0 | Clustered randomized sampling | General population | WHO | HOLOGIC | 6 |
| 23 | Li et al | 100.0 | Clustered randomized sampling | Hospital-based | WHO | USA SXA3000 | 8 |
| 24 | Hu et al | 100.0 | Clustered randomized sampling | Hospital-based | China | USA GE | 7 |
| 25 | Xu et al | N/A | Whole sample | Hospital-based | WHO | USA Norland | 7 |
| 26 | Zhao et al | 100.0 | Whole sample | Hospital-based | WHO | DXA | 6 |
| 27 | Yang et al | 92.2 | Whole sample | Hospital-based | WHO | USA GE | 8 |
| 28 | Li et al | 100.0 | Clustered randomized sampling | General population | WHO | Japan ALCKA | 7 |
| 29 | Mo et al | 100.0 | Clustered randomized sampling | General population | WHO | USA HOLOGIC | 8 |
| 30 | Wang et al | 100.0 | Whole sample | Hospital-based | WHO | Japan CM-200-181220 | 9 |
| 31 | Zhong et al | 96.5 | Whole sample | Hospital-based | China | France DMS | 7 |
| 32 | Wu et al | 100.0 | Whole sample | General population | WHO | Japan CM-200 | 8 |
| 33 | Ma et al | 100.0 | Clustered randomized sampling | General population | WHO | USA GE | 7 |
| 34 | Wang et al | 91.2 | Clustered randomized sampling | General population | China | Japan CM-200 | 8 |
| 35 | Yang et al | 100.0 | Whole sample | General population | WHO | France MEDILINK | 8 |
| 36 | Yi et al | 100.0 | Whole sample | General population | China | Israel SunlihTO-nisense700S | 8 |
| 37 | Pang et al | 100.0 | Clustered randomized sampling | General population | China | Korea EXA3000 | 8 |
| 38 | Ni et al | 100.0 | Whole sample | Hospital-based | China | Israel O-nisense700S | 5 |
| 39 | Wang et al | 98.7 | Whole sample | Hospital-based | WHO | N/A | 7 |
| 40 | Lan et al | 100.0 | Whole sample | General population | China | USA GE | 8 |
| 41 | Tu et al | 100.0 | Clustered randomized sampling | General population | WHO | France MEDILINK | 9 |
| 42 | Zhang et al | 100.0 | Clustered randomized sampling | General population | WHO | Japan CM-200 | 7 |
| 43 | Zhao et al | 100.0 | Whole sample | Hospital-based | China | France Medlink | 8 |
| 44 | Meng et al | N/A | Whole sample | Hospital-based | China | France Medlink | 7 |
| 45 | Miao et al | 85.9 | Clustered randomized sampling | General population | WHO | Israel O-nisense700S | 6 |
| 46 | Zhang et al | 100.0 | Whole sample | General population | China | German Somatom | 6 |
| 47 | Shen et al | 99.9 | Whole sample | Hospital-based | China | N/A | 8 |
| 48 | Li et al | 100.0 | Clustered randomized sampling | General population | WHO | France Medlink | 7 |
| 49 | Wu et al | N/A | Clustered randomized sampling | General population | WHO | USA GE | 7 |
| 50 | Sun et al | 99.3 | Clustered randomized sampling | General population | China | HOLOGIC | 8 |
| 51 | Shao et al | 99.1 | Whole sample | General population | WHO | France Medlink | 8 |
| 52 | Liu et al | 100.0 | Whole sample | General population | WHO | USA GE | 7 |
| 53 | Chen et al | 100.0 | Clustered randomized sampling | General population | China | USA DTX | 8 |
| 54 | Chen et al | 60.3 | Whole sample | General population | China | N/A | 9 |
| 55 | Mo et al | <100.0 | Clustered randomized sampling | General population | WHO | N/A | 7 |
| 56 | Liu et al | 81.1 | Clustered randomized sampling | General population | WHO | USA GE | 8 |
| 57 | Liang et al | 100.0 | Clustered randomized sampling | General population | China | China BH-8012 | 7 |
| 58 | Gao et al | 100.0 | Clustered randomized sampling | General population | China | France DEXA | 8 |
| 59 | Xu et al | 100.0 | Whole sample | Hospital-based | WHO | France DEXA | 8 |
| 60 | Wu et al | 100.0 | Whole sample | Hospital-based | China | USA DEXA | 7 |
| 61 | Chen et al | 100.0 | Clustered randomized sampling | General population | China | N/A | 8 |
| 62 | Yang et al | 87.2 | Clustered randomized sampling | General population | WHO | USA Lunar | 8 |
| 63 | Shuai et al | 100.0 | Stratified randomized sampling | General population | WHO | Japan Furono | 7 |
| 64 | Wang et al | 100.0 | Whole sample | Hospital-based | China | USA GE | 7 |
| 65 | Wan et al | 100.0 | Whole sample | General population | China | USA SPA | 6 |
| 66 | Wang et al | 100.0 | Clustered randomized sampling | General population | WHO | USA GE | 7 |
| 67 | Wang et al | 100.0 | Clustered randomized sampling | General population | WHO | USA CompuMed | 8 |
| 68 | Yang et al | 100.0 | Whole sample | General population | WHO | USA DTX200 | 8 |
| 69 | Li et al | N/A | Clustered randomized sampling | General population | WHO | France DMS | 7 |
